# 

**Published:** 1986-12

**Authors:** 


### BRISTOL MEDICO-CHIRURGICAL JOURNAL

VOLUME 101(vi) DECEMBER 1986

Appendicitis in Bristol 100 years ago
The Urological Opprobria
Learning about Haemophilia
The Bristol Eye Hospital
With Burton in Pokhara
Glass Paperweights
Poet's Corner
Current Comment and much else

NATIONAL LIBRARY
JUN 22 1987
OF MEDICINE

IN ASSOCIATION WITH

BRISTOL MEDICINE

THE MEDICAL JOURNAL OF THE SOUTH WEST

